# Quantum interference effects in multi-channel correlated tunneling structures

**DOI:** 10.1038/s41598-021-97199-2

**Published:** 2021-09-03

**Authors:** N. S. Maslova, V. N. Mantsevich, V. N. Luchkin, V. V. Palyulin, P. I. Arseyev, I. M. Sokolov

**Affiliations:** 1 Quantum Electronics Department, Quantum Technology Center, Faculty of Physics, Lomonosov Moscow State University, 119991 Moscow, Russia; 2 Department of Semiconductor Physics and Cryoelectronics, Quantum Technology Center, Faculty of Physics, Lomonosov Moscow State University, 119991 Moscow, Russia; 3 Department of Semiconductor Physics and Cryoelectronics, Faculty of Physics, Lomonosov Moscow State University, 119991 Moscow, Russia; 4 Center for Computational and Data-intensive Science and Engineering, Skolkovo Institute of Science and Technology, 121205 Moscow, Russia; 5 P.N. Lebedev Physical Institute of the Russian Academy of Science, 119991 Moscow, Russia; 6 Institut fur Physik, IRIS Adlesdorf, Humboldt Universitat zu Berlin, Newtonstrasse 15, 12489 Berlin, Germany

**Keywords:** Physics, Condensed-matter physics, Semiconductors, Quantum dots, Electronic properties and materials, Quantum dots

## Abstract

In multi-channel tunneling systems quantum interference effects modify tunneling conductance spectra due to Fano effect. We investigated the impact of Hubbard type Coulomb interaction on tunneling conductance spectra for the system formed by several interacting impurity atoms or quantum dots localised between the contact leads. It was shown that the Fano shape of tunneling conductance spectra strongly changes in the presence of on-site Coulomb interaction between localised electrons in the intermediate system. The main effect which determines the shape of the tunneling peaks could be not Fano interference but mostly nonequilibrium dependence of the occupation numbers on bias voltage.

## Introduction

Impurity atoms and quantum dots strongly affect the semiconductor electron transport and, thus, became promising candidates for both the implementation in semiconductor nanoelectronic devices^1–4^ and for the quantum transport phenomena investigation^5–11^. The individual atoms and quantum dots are convenient building blocks for nanoelectronics due to their stable well-defined electronic structure. Their integration in the semiconductor medium opens an opportunity for the final step in miniaturisation of electronics allowing fabrication of unique single-atom single-electron tunneling devices. The latter range from prototypes of quantum logic gates^12^ to quantum bits^13–15^ or charge pumps and turnstiles^16–19^. Moreover, the electrical properties of disordered nanomaterial systems allow to perform and advance reconfigurable computing. In Ref.^20^ a network of interconnected metal nanoparticles was shown to operate as interacting nonlinear single-electron transistors. It was found that the network can be adjusted in situ into any of the two-input Boolean logic gates. The proposed system meets the criteria for the physical realisation of (cellular) neural networks: universality (arbitrary Boolean functions), compactness, robustness and evolvability.

The impurity atoms and quantum dots being intermediate semiconductor nanoscale systems substantially modify the local electronic structure and consequently define the electron transport characteristics which can be studied using tunneling contacts^21–23^. Typically, experimental and theoretical investigations of multi-channel electron transport in these systems reveal Fano-type line shape in local tunneling conductance^24–27^. This Fano-type line shape appears due to the interference between the resonant transport through a quantum dot and a direct channel. Experimental investigations of both electronic structure and transport properties of impurity clusters or quantum dots systems can be carried out with the help of STM/STS technique^21^, while the conventional theoretical analysis uses methods such as Green’s functions formalism^28^, renormalisation group approach^29^, slave-boson mean-field theory^30^ or equations of motion^31^. Comparison of experimental results with theoretical calculations provides information whether electron transport occurs coherently or incoherently and gives an opportunity to determine the impurity type. The main effects are caused by local changes of the initial density of states due to interactions between nonequilibrium particles in the contact area. The Coulomb interaction of conduction electrons with nonequilibrium localised charges can result in nontrivial behaviour of tunneling characteristics. It is also important to note that the presence of Coulomb repulsion is a key factor influencing the energy spectrum and transport properties of intermediate systems formed by interacting impurity atoms or quantum dots^32,33^.

Since individual atoms or quantum dots-based electronic circuits are under active investigation, the understanding of both the role of interparticle interaction and quantum interference in such systems and the analysis of their influence on the electron transport properties is a necessary step on the way to the creation of single atom nanoelectronic devices. In the single electron regime such structures are proposed to be utilised as spin qubits^34,35^. In addition, such systems can be used as an effective spin filters^31,36^. The more complex double-dot structures are studied intensively as they are very promising for quantum interferometry and quantum computing^37,38^. In the systems formed by two or more quantum dots with Coulomb correlations the role of quantum interference effects still remains to be explored.

In the present paper we will concentrate on the crossover between the symmetry blockade regime and the regime when Fano effect arises for different ratio of tunneling coupling between the dots and the contact leads and on modification of multiple Fano resonances in the presence of Coulomb correlations. The Coulomb correlations between localised electrons are considered exactly without the use of the mean-field or non-crossing approximations. General expression describing tunneling conductance of the arbitrary multi-channel intermediate system was obtained and applied for the analysis of the electron transport properties for different spatial configurations of the impurity atoms. We avoid considering Kondo regime^39–41^ and Aharonov–Bohm oscillations^28,42–44^ since they were previously extensively studied. The effects were already scrutinised for a variety of geometries containing quantum dots such as side-coupled quantum dots^29,30,45^, the cases when a quantum dot is localised in each of the tunneling channels^46–48^, a chain of quantum dots localised in one of the channels^32,49^ and a double-dot geometry^50–52^.

The paper is organised as follows. The model Hamiltonian and the general expression for tunneling conductivity are described in “General case”. The electron transport occurring through two parallel channels each with impurity atoms is considered in “Two parallel tunneling channels: each channel with impurity atom”. The analysis of tunneling conductance peculiarities in the case when one of the channels for electron transports includes a chain of impurities and another one is a direct channel is given in “Direct channel and channel with a chain of impurities”. The role of Coulomb interaction is discussed in “The role of Coulomb interaction”. Conclusions are given in “Conclusion”.

## Tunneling through intermediate system

### General case

**Figure 1:**
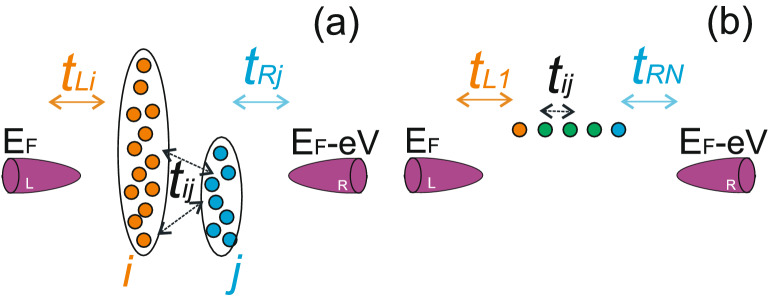
(Color online) Scheme of the tunneling contact with intermediate system formed by two clusters of impurity atoms (quantum dots). **(a)** General case of arbitrary intermediate system; **(b)** Intermediate chain-like structure.

Here we consider quantum transport through intermediate system formed by interacting impurity atoms or quantum dots localised between the tunneling contact leads (see Fig. 1a). The intermediate system is formed by the two clusters of impurity atoms (quantum dots) interacting with each other and with only one of the tunneling contact leads.

The Hamiltonian of the system consists of three parts, the intermediate system contribution ${\hat{H}}_0$, the tunneling processes between the intermediate system and the tunneling contact leads ${\hat{H}}_{tun}$ as well as electron states in the reservoir ${\hat{H}}_{res}$ (hereinafter we assume that $\hbar =1$ and $e=1$). The intermediate system Hamiltonian without Coulomb interaction reads

1 $$\begin{aligned} {\hat{H}}_0= & {} \sum _{i}\varepsilon _i{\hat{a}}_{i}^{\sigma \dag }{\hat{a}}_{i}^{\sigma }+\sum _{j}\varepsilon _j{\hat{a}}_{j}^{\sigma \dag }{\hat{a}}_{j}^{\sigma }+\sum _{ij}t_{ij}({\hat{a}}_{i}^{\sigma \dag }{\hat{a}}_{j}^{\sigma }+{\hat{a}}_{j}^{\sigma \dag }{\hat{a}}_{i}^{\sigma })\nonumber \\+ & {} \sum _{ii'}t_{ii'}({\hat{a}}_{i}^{\sigma \dag }{\hat{a}}_{i'}^{\sigma }+{\hat{a}}_{i'}^{\sigma \dag }{\hat{a}}_{i}^{\sigma })+\sum _{jj'}t_{jj'}({\hat{a}}_{j}^{\sigma \dag }{\hat{a}}_{j'}^{\sigma }+{\hat{a}}_{j'}^{\sigma \dag }{\hat{a}}_{j}^{\sigma }),\nonumber \\ \end{aligned}$$

where ${\hat{a}}_{i(j)}^{\sigma }$ is the electron annihilation operator for the single occupied localised state (site) with energy $\varepsilon _{i(j)}$ and spin $\sigma$ in each cluster and $t_{ij}$ is the tunneling transfer amplitude between sites *i* and *j* corresponding to different clusters. Sites *i* are directly coupled to the states in the lead *L* and sites *j* are connected with the states in the lead *R*. Hoppings between the impurities in each cluster are described by the tunneling transfer amplitudes $t_{ii'}$ and $t_{jj'}$.

Tunneling Hamiltonian ${\hat{H}}_{tun}$ has the following form:

2 $$\begin{aligned} {\hat{H}}_{tun}= & {} \sum _{ki}t_{Li}{\hat{c}}_{k}^{\sigma \dag }{\hat{a}}_{i}^{\sigma }+\sum _{pj}t_{Rj}{\hat{c}}_{p}^{\sigma \dag }{\hat{a}}_{j}^{\sigma }+h.c., \end{aligned}$$

where ${\hat{c}}_{k(p)}^{\sigma }$ is the electron annihilation operator for the electrons in the leads with energies $\varepsilon _{k(p)}$, spin $\sigma$ and quantum number *k*(*p*). $t_{Li(Rj)}$ is the tunneling transfer amplitude between the localised state and the tunneling contact leads. Furthermore, we assume that the tunneling transfer amplitudes $t_{Li(Rj)}$ have a negligibly weak dependence on *k*(*p*). Hence, the density of states in the leads is constant and the tunneling relaxation rates are constant as well. We treat the rates as parameters. Electron states in the leads of the tunneling contact are described by the Hamiltonian ${\hat{H}}_{res}$,

3 $$\begin{aligned} {\hat{H}}_{res}=\sum _{k}\varepsilon _k{\hat{c}}_{k}^{\sigma \dag }{\hat{c}}_{k}^{\sigma }+\sum _{p}(\varepsilon _p-eV){\hat{c}}_{p}^{\sigma \dag }{\hat{c}}_{p}^{\sigma }. \end{aligned}$$

Here the voltage *eV* applied to the contact is written explicitly as a shift of the chemical potential of one of the leads. Further in this section we will omit index $\sigma$ as it becomes important only in the presence of Coulomb interaction. Using Keldysh Green’s functions formalism^53^ one can get the following expression for the tunneling current flowing through the intermediate system formed by coupled impurity atoms or quantum dots,

4 $$\begin{aligned} I_{T}= & {} \sum _{k}\frac{\partial n_{k}}{\partial t}=\sum _{ik}t_{Li}(G_{ik}^{<}-G_{ki}^{<}), \end{aligned}$$

where the lesser Green’s function $$G_{ik}^{<}=i\langle a_{i}^{\dag }c_{k}\rangle $$ and $$ n_{k}$$ is the occupation number in the lead of tunneling contact. Taking into account relations between the lesser Green’s function $$G_{ik}^{<}$$ and the Green’s functions of intermediate system one can obtain the following expression for the tunneling current flowing through the intermediate system^40,54^:

5 $$\begin{aligned} I_{T}= & {} 2\int \frac{1}{2\pi } d\omega \sum _{ii'}(G_{i'i}^{A}(\omega )-G_{ii'}^{R}(\omega ))t_{Li}t_{Li'}\nu _{L}(\omega )n_{L}^{0}(\omega )- 2\int \frac{1}{2\pi } d\omega \sum _{ii'}t_{Li}t_{Li'}G_{ii'}^{<}(\omega )\nu _{L}(\omega ), \end{aligned}$$

where $n_{L}^{0}(\omega )$ is the equilibrium Fermi distribution for electrons in the tunneling contact lead *L* and $\nu _{L(R)}$ is the density of states in the leads of the tunneling contact. Tunneling contact leads are considered to be ideal wide band metals, so density of states is proportional to the inverse band width and considered to change slightly with energy, thus it can be considered to be a constant in all the calculations. Retarded (advanced) Green’s function corresponding to the intermediate system sites $G_{ii'}^{R(A)}(\omega )$ and lesser Green’s function $G_{ii'}^{<}(\omega )$ satisfy the following Dyson equations,

6 $$\begin{aligned}&G_{ii'}^{R(A)}(\omega )=G_{ii}^{0R(A)}(\omega )+G_{ii_{1}}^{0R(A)}(\omega )\Sigma _{i_{1}i_{2}}^{R(A)}(\omega )G_{i_{1}i'}^{R(A)}(\omega )+ G_{jj_{1}}^{0R(A)}(\omega )\Sigma _{j_{1}j_{2}}^{R(A)}(\omega )G_{j_{1}j'}^{R(A)}(\omega ),\nonumber \\&G_{ii'}^{<}(\omega )=G_{ii_{1}}^{R}(\omega )\Sigma _{i_{1}i_{2}}^{<}(\omega )G_{i_{1}i'}^{A}(\omega )+ G_{ij_{1}}^{R}(\omega )\Sigma _{j_{1}j_{2}}^{<}(\omega )G_{j_{2}i'}^{A}(\omega ), \end{aligned}$$

where unperturbed sites’ Green’s functions $G_{ii}^{0R(A)}(\omega )$ include all the electron transitions which can occur in the intermediate system. Lesser and retarded (advanced) self-energies read

7 $$\begin{aligned} \Sigma _{i_{1}i_{2}}^{<}(\omega )= & {} 2i\pi t_{Li_1}t_{Li_2}\nu _L(\omega )n_{L}^{0}(\omega ),\nonumber \\ \Sigma _{j_{1}j_{2}}^{<}(\omega )= & {} 2i\pi t_{Rj_1}t_{Rj_2}\nu _R(\omega )n_{R}^{0}(\omega ),\nonumber \\ \Sigma _{i_{1}i_{2}}^{R(A)}(\omega )= & {} \mp i\pi t_{Li_1}t_{Li_2}\nu _L(\omega ),\nonumber \\ \Sigma _{j_{1}j_{2}}^{R(A)}(\omega )= & {} \mp i\pi t_{Rj_1}t_{Rj_2}\nu _R(\omega ). \end{aligned}$$

The following relations for Green’s functions and self-energies are valid in the stationary case

8 $$\begin{aligned} G^{R}(\omega )[\Sigma ^{A}(\omega )-\Sigma ^{R}(\omega )] G^{A}(\omega )=G^{A}(\omega )-G^{R}(\omega ). \end{aligned}$$

After substitution of Eqs. (6)–(8) into Eq. (5) the terms with self-energies $\Sigma _{i_{1}i_{2}}^{<}(\omega )$ in the lesser Keldysh Green’s functions $G_{ii'}^{<}(\omega )$ exactly cancel the first term in the right hand side in Eq. (5) for the tunneling current due to the validity of the relation (8). Finally one can get the expression, which describes the electron transport between the tunneling contact leads through the arbitrary intermediate system formed by the system of impurity atoms or quantum dots. This expression is a generalisation of that obtained in^55^ for a single atomic chain:

9 $$\begin{aligned} I_{T}= & {} 4\int \frac{1}{2\pi } d\omega \sum _{ii'j_1j_2}\nu _{L}(\omega )t_{Li}G_{ij_1}^{R}(\omega )t_{Rj_1}\nu _{R}t_{j_2R}G_{j_{2}i'}^{A}(\omega )t_{Li'}\times [n_{L}^{0}(\omega )-n_{R}^{0}(\omega )]. \end{aligned}$$

The formula (9) in a correct way takes into account not only contributions from all possible trajectories of electron transport through the intermediate system but also from all interactions inside the intermediate system.

### Two parallel tunneling channels: each channel with impurity atom

**Figure 2:**
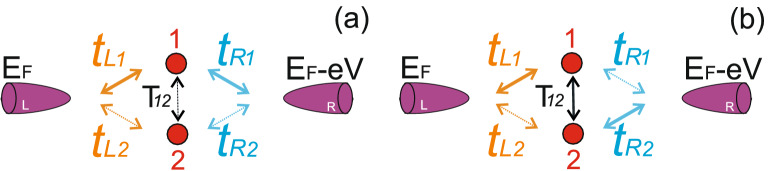
(Color online) Schemes of tunneling between tunneling contact leads and intermediate system formed by two interacting impurities. Changing the ratio between various tunneling amplitudes one gets different regimes. **(a)** Shows the regime when tunneling between the leads through one of the impurity atoms strongly exceeds tunneling between the leads through the other one, $t_{L1},t_{R1}>>t_{L2},t_{R2}$; **(b)** demonstrates the regime when tunneling between the impurity atoms is strong and $t_{L1},t_{R2}>>t_{L2},t_{R1}$. This is the case of tunneling through two-atom chain. Solid lines demonstrate the main tunneling channels. Dashed lines show the weak tunneling channels. Tunneling between the impurity atoms is described by the amplitude $T_{12}$.

Now we can use the expression (9) to describe the electron transport through the intermediate system formed by the two interacting impurity atoms each coupled to both tunneling contact leads in the absence of Coulomb interaction (see Fig.2a). The electron transport occurs through two parallel channels, each with an impurity atom. Transitions between the impurities are also present and are described by the tunneling transfer amplitude $T_{12}$. In the presence of the tunneling between the impurities retarded Green’s functions in Eq. (9) are:

10 $$\begin{aligned} G_{11}^{R}(\omega )= & {} \frac{\omega -\varepsilon _2+i\Gamma _2}{(\omega -\varepsilon _1+i\Gamma _1)(\omega -\varepsilon _2+i\Gamma _2)-T_{12}^{2}},\nonumber \\ G_{22}^{R}(\omega )= & {} \frac{\omega -\varepsilon _1+i\Gamma _1}{(\omega -\varepsilon _1+i\Gamma _1)(\omega -\varepsilon _2+i\Gamma _2)-T_{12}^{2}},\nonumber \\ G_{12}^{R}(\omega )= & {} \frac{T_{12}}{(\omega -\varepsilon _1+i\Gamma _1)(\omega -\varepsilon _2+i\Gamma _2)-T_{12}^{2}}, \end{aligned}$$

where $\Gamma _1=\Gamma _{L1}+\Gamma _{R1}$ and $\Gamma _2=\Gamma _{L2}+\Gamma _{R2}$ and $G_{ij}^{A}(\omega )=[G_{ij}^{R}(\omega )]^{*}$ and tunneling transfer rates $\Gamma _{Li(Rj)}=\pi \nu _{L(R)}t_{Li(Rj)}^{2}$ with $\nu _{L(R)}$ being the density of states in the leads of the tunneling contact. Furthermore, all the expressions are given for the general case $\varepsilon _1\ne \varepsilon _2$, but numerical calculations for simplicity will be performed for the resonant case when single electron energy levels of both impurity atoms have the same value $\varepsilon _1=\varepsilon _2$.

**Figure 3:**
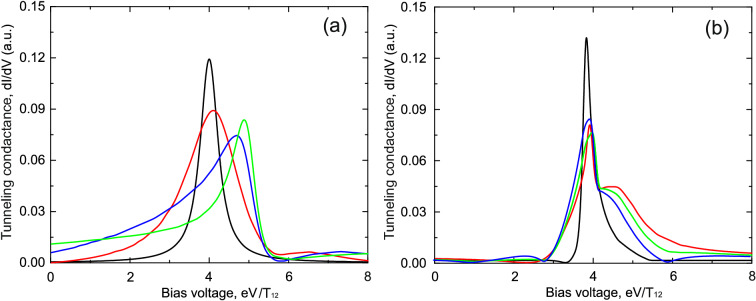
(Color online) Tunneling conductance as a function of applied bias voltage for the impurities configuration shown in Fig.2a. **(a)** Demonstrates results in the absence of Coulomb interaction; **(b)** shows calculation results in the presence of infinitely large Coulomb interaction. Black curves correspond to the tunneling rates $t_{L1}=0.5T_{12}$, $t_{R1}=0.5T_{12}$, $t_{L2}=0.5T_{12}$, $t_{R2}=0.5T_{12}$; red curves correspond to the tunneling rates $t_{L1}=0.8T_{12}$, $t_{R1}=0.8T_{12}$, $t_{L2}=0.5T_{12}$, $t_{R2}=0.5T_{12}$; blue curves correspond to the tunneling rates $t_{L1}=1.2T$, $t_{R1}=1.2T_{12}$, $t_{L2}=0.5T_{12}$, $t_{R2}=0.5T_{12}$; green curves correspond to the tunneling rates $t_{L1}=1.5T_{12}$, $t_{R1}=1.5T_{12}$, $t_{L2}=0.5T_{12}$, $t_{R2}=0.5T_{12}$. Parameters $\varepsilon _1=\varepsilon _2=5.0T_{12}$, $T_{12}=1$ are the same for all figures.

**Figure 4:**
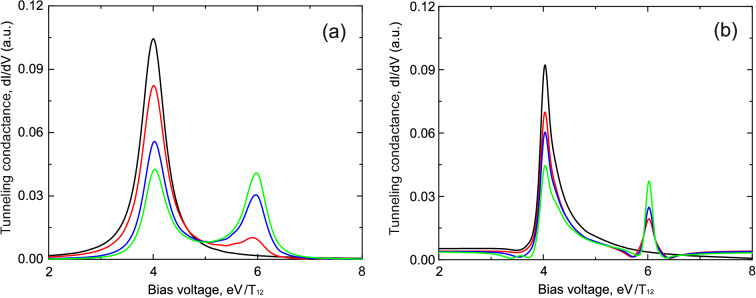
(Color online) Tunneling conductance as a function of applied bias voltage for the impurities configuration shown in Fig.2b. **(a)** Demonstrates results in the absence of Coulomb interaction; **(b)** shows calculation results in the presence of infinitely large Coulomb interaction. Black curves correspond to the tunneling rates $t_{L1}=0.4T_{12}$, $t_{R1}=0.4T_{12}$, $t_{L2}=0.4T_{12}$, $t_{R2}=0.4T_{12}$; red curves correspond to the tunneling rates $t_{L1}=0.4T_{12}$, $t_{R1}=0.15T_{12}$, $t_{L2}=0.15T_{12}$, $t_{R2}=0.4T_{12}$; blue curves correspond to the tunneling rates $t_{L1}=0.4T_{12}$, $t_{R1}=0.04T_{12}$, $t_{L2}=0.04T_{12}$, $t_{R2}=0.4T_{12}$; green curves correspond to the tunneling rates $t_{L1}=0.4T_{12}$, $t_{R1}=0.002T_{12}$, $t_{L2}=0.002T_{12}$, $t_{R2}=0.4T_{12}$. Parameters $\varepsilon _1=\varepsilon _2=5.0T_{12}$, $T_{12}=1$ are the same for all figures.

The most interesting regimes in the considered system occur in following two cases of coupling between the impurity atoms and the leads: 1. The “parallel” regime when tunneling between the leads through one of the impurity atoms strongly exceeds tunneling between the leads through the another one ($t_{L1},t_{R1}>>t_{L2},t_{R2}$, see Fig. 2a). In this case electron transport occurs through two parallel channels but one of the tunneling channels dominates; 2. The “sequential” regime when the sequential tunneling channel is a dominating one ($t_{L1},t_{R2}>>t_{L2},t_{R1}$, see Fig. 2b). In this case electron transport occurs through a chain formed by two impurity atoms each of them mostly coupled to one of the leads. Calculation results for both tunneling regimes are shown in Figs. 3 and 4.

For both regimes in the case of symmetric tunneling contact when all tunneling amplitudes between the impurity atoms and the contact leads have the same values ($t_{L1}=t_{R1}=t_{L2}=t_{R2}$) only a single peak with a symmetric shape is present in the tunneling conductance corresponding to the symmetric state of the two impurities complex with the energy $\varepsilon -T$ (see the black curves in Figs. 3a and 4a). Peak corresponding to the antisymmetric state with energy $\varepsilon +T$ is damped due to the destructive interference. The presence of only one well pronounced peak in tunneling conductance is the direct manifestation of symmetry blockade which results in the destructive interference. If one of the tunneling channels prevails in electron transport through the impurity complex ($t_{L1},t_{R1}>>t_{L2},t_{R2}$), the single peak corresponding to the symmetric state begins to change its shape to the asymmetric one (see the red, the blue and the green curves in Fig. 3a). The asymmetry of the peak’s shape is the most pronounced when tunneling through one of the channels is nearly absent (see the green curve in Fig. 3a). Further it will be demonstrated that tunneling conductance behavior considerably changes in the presence of Coulomb interaction (see Fig. 3b). Most pronounced changes occur in the vicinity of the single electron energy levels or when applied bias is close to the energy of electron transitions between electronic states with *n* and $n+1$ electrons. In the regime when the sequential tunneling between   the impurity atoms becomes important ($t_{L1},t_{R2}>>t_{L2},t_{R1}$, see Fig.  4a) the second peak corresponding to the antisymmetric state appears. The amplitude of the symmetric peak decreases and the amplitude of the antisymmetric peak increases with the growth of the asymmetry between the tunneling amplitudes (see the red, the blue and the green curves in Fig. 4a). When the tunneling amplitudes $t_{L2},t_{R1}$ become negligibly small the amplitudes of two peaks become very close to each other (see the green curve in Fig. 4a). Thus, changing the anisotropy of the kinetic processes one can observe the crossover between the symmetry blockade regime and the regime when Fano asymmetrical peak arises if the difference of values of hopping integrals is substantial. To conclude, in this section we analysed in details the crossover between the regime of resonant tunneling through the symmetric single electron state of double QDs in the case of parallel tunneling to the regime when Fano effect becomes well resolved. Fano effect appears when tunneling rates between one of the QDs and the leads strongly exceed tunneling rate for another QD (see Fig. 3a). We also studied the tunneling conductance modification, which is a result of transfer from the regime of parallel tunneling to the regime of sequential tunneling (see Fig. 4a). In this case one can follow the appearance of double peak structure in tunneling conductance. The obtained results open the possibility to choose the proper experimental geometry to get a required regime of electron transport and corresponding properties of tunneling conductance spectra.

### Direct channel and channel with a chain of impurities

**Figure 5:**
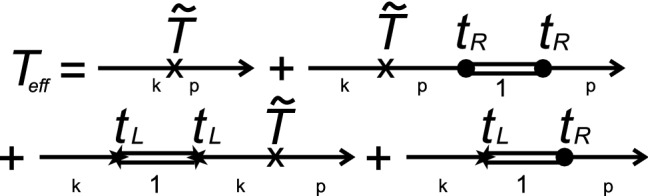
(Color online) Diagrams contributing to the effective tunneling transfer amplitude $T_{eff}$ considering multiple electrons returns from intermediate site to one of the contact leads.

In the case when the intermediate system is formed by a chain of impurities, one of the tunneling channels corresponds to the electron transitions between the leads through the chain and another one is a direct channel between the tunneling contact leads. If the intermediate system is an atomic chain consisting of *N* sites (see Fig. 1b), one can get a rather simple expression for tunneling current using Eq. (9)^55^,

11 $$\begin{aligned} I_{T}= & {} 4\int \frac{1}{2\pi } d\omega \Gamma _{L1}\Gamma _{RN}|G_{1N}^{R}(\omega )|^{2}[n_{L}^{0}(\omega )-n_{R}^{0}(\omega )],\nonumber \\ \end{aligned}$$

where $\Gamma _{L1(RN)}=\pi \nu _{L(R)}t_{L1(RN)}^{2}$ is the tunneling transfer rate. In this case one can consider $\Gamma _{L1}\Gamma _{RN}|G_{1N}^{R}(\omega )|^{2}$ as an effective transmission amplitude $|T_{eff}|^{2}$. Direct tunneling between the leads is described by the Hamiltonian ${\hat{H}}_{direct}$,

12 $$\begin{aligned} {\hat{H}}_{direct}={\tilde{T}}\sum _{k,p}({\hat{c}}_{k}^{\sigma \dag }{\hat{c}}_{p}^{\sigma }+{\hat{c}}_{p}^{\sigma \dag }{\hat{c}}_{k}^{\sigma }), \end{aligned}$$

where the tunneling transfer amplitude ${\tilde{T}}$ is assumed to be independent of momentum and spin. Taking into account the interference effects between two channels one can get the general expression for the effective transmission amplitude $T_{eff}$ which can be represented by the diagrams shown in Fig. 5 and has the form

13 $$\begin{aligned} T_{eff}(\omega )= & {} {\tilde{T}}[1+i\Gamma _{L1}G_{11}^{R}(\omega )+i\Gamma _{RN}G_{NN}^{R}(\omega )]+\sqrt{\Gamma _{L1}\Gamma _{RN}}G_{1N}^{R}(\omega ), \end{aligned}$$

where $G_{jj}^{R}$ is the retarded Green’s function of the *j*-th impurity in the chain and *N* is a number of impurities in the chain.

Expression (13) is the main result of this section. The effective transmission amplitude (Eq. 13) takes into account multiple electron transitions between the lead and the intermediate system (impurity atom or quantum dot). It describes transmission through the direct channel and through the intermediate system as well as interference contributions. Expression (13) describes both the inter-channel and the intra-channel interference. The first term corresponds to the inter-channel interference, which occurs due to the presence of several paths of electron transport. The second term describes the intra-channel interference and takes into account the interference between any trajectory due to the multiple electron transitions between the lead and the intermediate system calculated using nonequilibrium Keldysh diagram technique. For completeness the simple case with a single impurity localised in one of the channels is described in Section 1 of the Supplementary Material.

**Figure 6:**
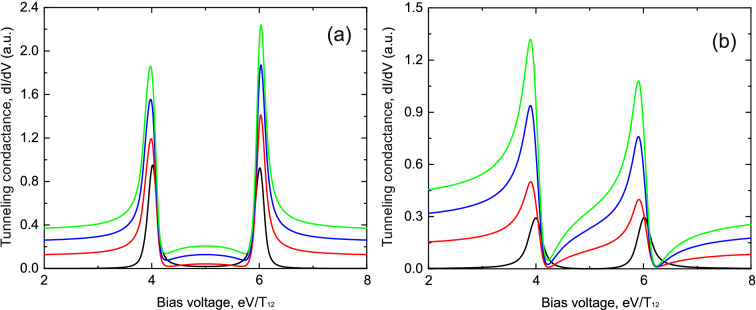
(Color online) Tunneling conductance as a function of applied bias voltage for the impurities configuration shown in Fig. 3b in the case of the intermediate system formed by a chain of two impurity atoms. **(a)** Demonstrates results in the absence of Coulomb interaction; **(b)** shows calculation results in the presence of infinitely large Coulomb interaction. Black curves correspond to the tunneling rates $t_{L1}=t_{R2}=0.3T_{12}$, $T=0.0$; red curves correspond to the tunneling rates $t_{L1}=t_{R2}=0.3T_{12}$, $T=0.6T_{12}$; blue curves correspond to the tunneling rates $t_{L1}=t_{R2}=0.3T_{12}$, $T=1.0T_{12}$; green curves correspond to the tunneling rates $t_{L1}=t_{R2}=0.3T_{12}$, $T=1.2T_{12}$. Parameters $\varepsilon _1=\varepsilon _2=5.0T_{12}$, $T_{12}=1$ are the same for all figures.

**Figure 7:**
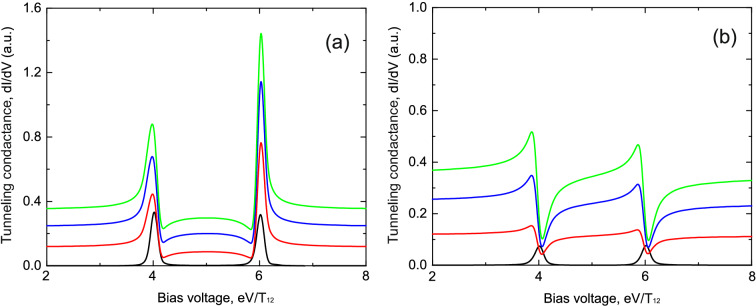
(Color online) Tunneling conductance as a function of applied bias voltage for the impurities configuration shown in Fig. 3b in the case of the intermediate system formed by a chain of two impurity atoms. **(a)** Demonstrates results in the absence of Coulomb interaction; **(b)** shows calculation results in the presence of infinitely large Coulomb interaction. Black curves correspond to the tunneling rates $t_{L1}=0.1T$, $t_{R2}=0.3T_{12}$, $T=0.0$; red curves correspond to the tunneling rates $t_{L1}=0.1T$, $t_{R2}=0.3T_{12}$, $T=0.6T_{12}$; blue curves correspond to the tunneling rates $t_{L1}=0.1T$, $t_{R2}=0.3T_{12}$, $T=1.0T_{12}$; green curves correspond to the tunneling rates $t_{L1}=0.1T$, $t_{R2}=0.3T_{12}$, $T=1.2T_{12}$. Parameters $\varepsilon _1=\varepsilon _2=5.0T_{12}$, $T_{12}=1$ are the same for all figures.

When the atomic chain consists of two impurities we get for the retarded Green’s functions

14 $$\begin{aligned} G_{11}^{R}(\omega )= & {} \frac{\omega -\varepsilon _1}{(\omega -\varepsilon _1+i\Gamma _{L1})(\omega -\varepsilon _{2}+i\Gamma _{R2})-T_{12}^{2}},\nonumber \\ G_{22}^{R}(\omega )= & {} \frac{\omega -\varepsilon _2}{(\omega -\varepsilon _1+i\Gamma _{L1})(\omega -\varepsilon _{2}+i\Gamma _{R2})-T_{12}^{2}},\nonumber \\ G_{12}^{R}(\omega )= & {} \frac{T_{12}}{(\omega -\varepsilon _1+i\Gamma _{L1})(\omega -\varepsilon _{2}+i\Gamma _{R2})-T_{12}^{2}}, \end{aligned}$$

where $T_{12}$ is the tunneling transfer amplitude between the impurity atoms in the chain. Substituting the Green’s functions in (13) one can calculate tunneling conductance (neglecting Coulomb interaction) which is shown in Figs. 6 and 7. Figure 6 corresponds to the symmetric coupling of impurities chain with the leads of the tunneling contact ($t_{L1}=t_{R2}$), while Fig. 7 shows the case of the asymmetric coupling ($t_{L1}\ne t_{R2}$). It demonstrates two well resolved peaks corresponding to the symmetric $\varepsilon +T$ and antisymmetric $\varepsilon -T$ states of the impurity complex. Both peaks have Fano-like asymmetric shape and the asymmetry increases with the growth of direct tunneling transition amplitude since in this case interference effects in the system become more pronounced. The presence of asymmetry is a direct consequence of both the constructive intra-channel and inter-channel interference. Proposed approach can easily be generalised for the case of more than two impurity atoms in the chain.

## The role of Coulomb interaction

Now we take into account Coulomb interaction between the electrons localised in the intermediate system. We will consider on-site Coulomb repulsion of electrons localised in the same impurity atom and neglect for simplicity inter-site Coulomb repulsion between the electrons localised in the different impurity atoms. Let us remind that we do not take into account interaction between localised electrons and band electrons in the leads as Kondo regime is beyond the scope of this work. Thus, the interaction Hamiltonian reads

15 $$\begin{aligned} {\hat{H}}_{int}=\sum _{i}U_{i}{\hat{n}}_{i}^{\sigma }{\hat{n}}_{i}^{-\sigma }, \end{aligned}$$

where ${\hat{n}}_{i}^{\sigma }={\hat{a}}_{i}^{\sigma +}{\hat{a}}_{i}^{\sigma }$ and $U_{i}$ is the on-site Coulomb repulsion and *i* is the number of the impurity atom or quantum dot. To take Coulomb interaction into account one can use the expressions for the tunneling conductance obtained in the previous sections with the corresponding modification of Green’s functions. Now retarded and advanced functions depend on electron occupation numbers $n_i$ which should be determined from the calculations. For brevity we present here only the case of very large Coulomb interaction *U*, i.e. when it strongly exceeds the single electron energies and the applied bias voltage. That means that only one electron with a fixed spin projection can be localised at the impurity energy level^56^.

### Two parallel tunneling channels: each channel with impurity atom

In the case of large Coulomb interaction Green’s functions look as^57^

16 $$\begin{aligned} G_{1}^{R}(\omega )= & {} \frac{1-n_1-2n_2}{\omega -\varepsilon _1+i\Gamma _1},\nonumber \\ G_{2}^{R}(\omega )= & {} \frac{1-n_2-2n_1}{\omega -\varepsilon _2+i\Gamma _2}. \end{aligned}$$

Section 3 of the Supplementary Material shows how to calculate nonequilibrium occupation numbers $n_1$ and $n_2$ for the impurity atoms states. Using expressions (13) and (16) one can calculate tunneling conductance in the presence of strong Coulomb interaction. Results for the both “parallel” and “sequential” tunneling regimes are shown in Figs. 3b and 4b, correspondingly. As it was mentioned above in “Two parallel tunneling channels: each channel with impurity atom” the difference between the tunneling regimes is caused by different tunneling amplitudes ratio. In both cases Coulomb interaction strongly modifies the shape of the peaks in tunneling conductance. For the case of “parallel” tunneling (Fig. 3b) asymmetric shape is “inverted” due to Coulomb correlation effects. For the “sequential” case (Fig. 4b) the asymmetry becomes much more pronounced. The asymmetry of the peaks is the direct manifestation of the tunneling conductivity modification by the occupation numbers which reveal the presence of Coulomb interaction in the system. In the regime of “parallel” tunneling (Fig. 3b) the presence of Coulomb interaction also leads to the “shoulders” for the tunneling conductance peak. The described behaviour of the conductance is determined by the corresponding features of the occupation numbers. The appearance of the “shoulders” in the presence of Coulomb interaction is a result of the dependence of tunneling probability on occupation numbers. Occupation numbers are changing strongly when applied bias is close to the single electron energy levels or to the energy of electron transitions between electronic states with *n* and $n+1$ particles. For low temperatures and small tunneling rates (tunneling rates are much smaller than all the electronic energies) the occupation numbers as a function of applied bias reveal a step like behavior. Each step appears when applied bias is equal to the energy of electron transitions between electronic states with *n* and $n+1$ particles. Such sharp change of occupation numbers leads to modification of tunneling conductance and results in the appearance of the “shoulders” in the vicinity of the resonant peak in tunneling conductance.

### Direct channel and channel with a chain of impurities

If tunneling occurs through a chain of two impurities (quantum dots) it is convenient to use symmetric and antisymmetric states as a basis set. Corresponding Green’s functions $G_{a}^{R}$ and $G_{s}^{R}$ can be written similar to Eq. (16) as^57^:

17 $$\begin{aligned} G_{a}^{R(A)}=\frac{1-n_{s}-2n_{a}}{\omega -\varepsilon _s\pm i\Gamma _a},\nonumber \\ G_{s}^{R(A)}=\frac{1-n_{a}-2n_{s}}{\omega -\varepsilon _a\pm i\Gamma _s} \end{aligned}$$

with $$\varepsilon _{s(a)}=\varepsilon \pm T_{12}$$ and $$\Gamma _{a(s)}=\Gamma _{La(s)}+\Gamma _{Ra(s)}$$.

For strong tunneling coupling between the impurities one could omit the last term in Eq. (13) after substitution of $G_{a}^{R}$ and $G_{s}^{R}$ into the formula for the tunneling current.

Expressions for occupation numbers $n_{s}$ and $n_{a}$ can be found from the stationary solution of kinetic equations similar to Eq. (5) in Sect. 3 of the Supplementary Material:

18 $$\begin{aligned} n_{s}=\frac{N_{s}^{T}(\varepsilon _s)(1-N_{a}^{T}(\varepsilon _a))}{[1+N_{s}^{T}(\varepsilon _s)][1+N_{a}^{T}(\varepsilon _a)]-4N_{s}^{T}(\varepsilon _s)N_{a}^{T}(\varepsilon _a)},\nonumber \\ n_{a}=\frac{N_{a}^{T}(\varepsilon _a)(1-N_{s}^{T}(\varepsilon _s))}{[1+N_{s}^{T}(\varepsilon _s)][1+N_{a}^{T}(\varepsilon _a)]-4N_{s}^{T}(\varepsilon _s)N_{a}^{T}(\varepsilon _a)},\nonumber \\ \end{aligned}$$

where

19 $$\begin{aligned} N_{a(s)}^{T}(\varepsilon _{a(s)})=\frac{\Gamma _{La(s)}N_{L}(\varepsilon _{a(s)})+\Gamma _{Ra(s)}N_{R}(\varepsilon _{a(s)})}{\Gamma _{La(s)}+\Gamma _{Ra(s)}}\nonumber \\ \end{aligned}$$

and

20 $$\begin{aligned} N_{L(R)}(X)=\frac{1}{\pi }\int d\omega n_{L(R)^{0}}(\omega )\frac{\Gamma _{La(s)}+\Gamma _{Ra(s)}}{(\omega -X)^{2}+(\Gamma _{La(s)}+\Gamma _{Ra(s)})^{2}},\nonumber \\ \end{aligned}$$

where $X=\varepsilon _{a,s}$ and for resonant tunneling $\Gamma _{a(s)}=1/2\Gamma _{1(2)}$.

Results for the tunneling conductivity in this case for symmetric and asymmetric coupling of the chain with the leads are shown in Figs. 6b and 7b. As in Figs. 3 and 4 Coulomb interaction strongly modifies the shape of the peaks which arise in the tunneling conductance. Modified peaks (Figs. 6b and 7b)reveal well resolved asymmetric Fano-like shape even in the case of small tunneling amplitude through the direct tunneling channel. We see that the asymmetry of peaks in this case reflects mostly the impact of Coulomb interaction on impurity states occupation numbers.

Let us now discuss the parameters and relations achievable in the real QDs systems. QDs parameters such as single electron energy levels, Coulomb interaction, tunneling amplitudes and coupling between the dots depend on the dot sizes and shapes as well as the growth procedure. These quantities determine the arrangement in space and, consequently, the strength of interaction of the dots with other dots and the leads. Typically, the tunneling amplitudes between the dots exceed the coupling between the dots and the tunneling contact leads. Single electron energy level values are larger than the tunneling amplitudes between the quantum dots. Coulomb correlation energies could exceed all other characteristics energy values in the system. For example, for *GaAs*/*AlGaAs*/*InGaAs* quantum dots^58–60^ the single electron energy level values are about 2–5 meV, $T_{12}$ is about 0.4 meV, Coulomb interaction is about 4–6 meV and coupling to the leads is about 10–100 $\upmu$eV. Similar values of the system parameters can be achieved for the Si-based quantum dots^61,62^.

## Conclusion

We investigated the conductance features for the system formed by several interacting impurity atoms or quantum dots localised between the leads. The derived generalised expression for effective tunneling transmission amplitude through multi-channel intermediate system allowed to evaluate the role of various interference effects. It was shown that crossover from Fano regime with an asymmetric peak in the tunneling conductivity to symmetry blockade regime with a single symmetric peak could be observed if one tunes the ratios between the tunneling rates (for example by external gate voltage ). The modification of tunneling conductivity spectra from “parallel” to “sequential” coupling to the leads was analysed in the frame of suggested approach.

On-site Coulomb interaction between localised electrons strongly modifies the effective tunneling probability, which depends on the nonequilibrium electron occupation numbers of the QDs. The double occupation of QDs states is restricted in a particular range of applied bias. Moreover, Coulomb interaction substantially changes the single electron interference picture due to the Coulomb correlations between tunneling electrons. The shape of tunneling conductance peaks is very sensitive to the geometry of the QDs system and the strength of Coulomb correlations. It was shown that in some cases the main effect which determines the shape of the tunneling peaks is not Fano interference but mostly nonequilibrium correlation effects for the occupation numbers of localised states.

## Supplementary Information

Supplementary Information.
