# Supplementary material for: Quantum interference effects in multi-channel correlated tunneling structures
Source: Sci Rep. 2021 Sep 3;11:17676. doi: 10.1038/s41598-021-97199-2 (PMC8417284; doi:10.1038/s41598-021-97199-2)
Supplement: Supplementary file 1 — Supplementary Information. [file 41598_2021_97199_MOESM1_ESM.pdf]

Supplementary material: Quantum interference effects in multi-channel correlated tunneling structures

N. S. Maslova[1,+], V. N. Mantsevich[2,*,+], V. N. Luchkin[3,+], V. V. Palyulin[4,+], P. I. Arseyev[5,+], and I. M. Sokolov[6,+]

[1]Quantum Technology Center and Quantum electronics department, Faculty of Physics, Lomonosov Moscow State University, 119991 Moscow, Russia
[2]Quantum Technology Center and department of Semiconductor physics and Cryoelectronics, Faculty of Physics, Lomonosov Moscow State University, 119991 Moscow, Russia
[3]Department of Semiconductor physics and Cryoelectronics, Faculty of Physics, Lomonosov Moscow State University, 119991 Moscow, Russia
[4]Center for Computational and Data-intensive Science and Engineering, Skolkovo Institute of Science and Technology, 121205, Moscow, Russia
[5]P.N. Lebedev Physical Institute of the Russian Academy of Science, 119991 Moscow, Russia, Moscow, Russia
[6]Institut fur Physik and IRIS Adlesdorf, Humboldt Universitat zu Berlin, Newtonstrasse 15, 12489 Berlin, Germany

ABSTRACT

Here we put some addition analytical derivations

1 Two parallel tunneling channels: direct channel and channel with a single impurity

In the situation when intermediate system is simply a single impurity expression for effective transmission amplitude (13) can be re-written in the following form:

$$T_{eff}(\omega)=\tilde{T}[1+i\Gamma G^R_{11}(\omega)]+\sqrt{\Gamma_{L1}\Gamma_{R1}}G^R_{11}(\omega), \tag{1}$$

where $G^R_{11}(\omega)=\frac{1}{\omega-\varepsilon\pm i(\Gamma_{L1}+\Gamma_{R1})}$, $\Gamma=\Gamma_{L1}+\Gamma_{R1}$ and with ε being the single electron energy level of the impurity. So, tunneling current can be obtained using Keldysh diagram technique with the effective transparency of the system $|T_{eff}(\omega)|^2$ considering multiple return processes

$$I_T(eV) \quad = \quad 4\int\frac{1}{2\pi}d\omega|T_{eff}(\omega)|^2[n^0_L(\omega)-n^0_R(\omega)], \tag{2}$$

where $|T_{eff}(\omega)|^2$ is given by the analytical expression considering retarded and advanced Green's functions of the impurity

$$\begin{aligned}|T_{eff}(\omega)|^2= \quad & \tilde{T}^2[1-\Gamma^2G^R_{11}(\omega)G^A_{11}(\omega)+y^2\Gamma^2G^R_{11}(\omega)G^A_{11}(\omega)+2y\Gamma ReG^R_{11}(\omega)]=\\ & \tilde{T}^2[1+\frac{2yx+y^2}{x^2+1}-\frac{1}{x^2+1}]=\tilde{T}^2\frac{(y+x)^2}{1+x^2}\end{aligned}$$

with parameters x, y, $\tilde{T}^2$ and Γ being

$$\begin{aligned}x &= \frac{\omega-\varepsilon_1}{\Gamma},\\ y^2 &= \frac{\Gamma_{L1}\Gamma_{R1}}{\tilde{T}^2\Gamma^2},\\ \tilde{T}^2 &= \frac{\pi^2T^2\nu_L\nu_R}{1+T^2\pi^2\nu_L\nu_R}.\end{aligned} \tag{3}$$

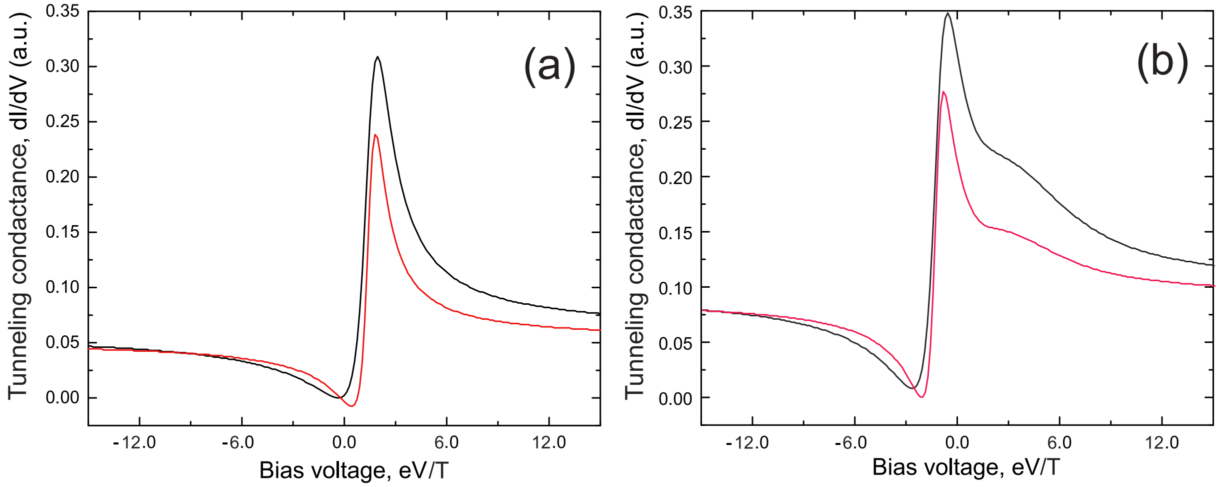

Figure 1. (Color online) Tunneling conductance as a function of applied bias voltage in the case when tunneling occurs through two channels: the direct channel and the resonant channel with a single impurity. Panel (a) corresponds to the calculations performed without Coulomb interaction $U = 0$; Panel (b) shows results obtained in the presence of Coulomb interactions. Black curves are obtained for the symmetric tunneling contact and red curves correspond to the asymmetric contact. Parameters $T = 1$; $\varepsilon = 1.5T$ are the same for all the figures. For black curves $\Gamma_{L1} = \Gamma_{R1} = 0.5T$, for red curves $\Gamma_{L1} = 0.5T$ and $\Gamma_{R1} = 0.15T$.

Panel (a) in Fig.1 shows tunneling conductance as a function of applied bias obtained in the absence of Coulomb interaction. Calculation results clearly reveal the well known Fano shape of the peak in tunneling conductance[1] both for symmetric ($\Gamma_{L1} = \Gamma_{R1}$) and asymmetric ($\Gamma_{L1} \neq \Gamma_{R1}$) tunneling contacts which arise due to the constructive interference between resonant tunneling channel through impurity state and direct tunneling channel between the contact leads.

2 Coulomb interaction in the case of two parallel tunneling channels: direct channel and channel with a single impurity

For the single impurity localised in the tunneling contact the retarded (advanced) impurity Green's function reads[2]:

$$G_{11}^{R(A)}(\omega) = \frac{1-n_1}{\omega - \varepsilon \pm i\Gamma}. \tag{4}$$

One can obtain kinetic equations for the averaged impurity occupation numbers $n_1^{\pm\sigma}(t) = \langle \hat{n}_1^{\pm\sigma}(t) \rangle$[3]

$$\dot{n}_1^{\pm\sigma}(t) = \Gamma[n_1^{\pm\sigma} - (1 - n_1^{\mp\sigma})N^T], \tag{5}$$

where occupation function N^T depends on the leads properties and have the form

$$N^T = \frac{\Gamma_{L1}}{\Gamma} N_L + \frac{\Gamma_{R1}}{\Gamma} N_R, \tag{6}$$

with function $N_{L(R)}$ being

$$N_{L(R)} = \frac{1}{2\pi} i \int d\varepsilon_{L(R)} n_{L(R)}^0(\varepsilon_{L(R)}) \left[\frac{1}{\varepsilon_1 + i\Gamma - \varepsilon_{L(R)}} - \frac{1}{\varepsilon_1 - i\Gamma - \varepsilon_{L(R)}} \right]. \tag{7}$$

The stationary solution of kinetic equation (5) determines the nonequilibrium occupation of the impurity,

$$n_1 = n_1^{\sigma} = n_1^{-\sigma} = \frac{N^T}{1+N^T}. \tag{8}$$

After substitution Eqs.(7) and (6) in expression (8) and considering the following relations $((1-\hat{n}_1^{\pm\sigma})\hat{n}_1^{\pm\sigma}=0$, $(\hat{n}_1^{\pm\sigma})^2=\hat{n}_1^{\pm\sigma})$ valid for fermions one can get an expression for the effective transmission amplitude $|T_{eff}(\omega)|^2$:

$$|T_{eff}(\omega)|^2 = |T_{eff}(\omega)|^2(1-n_1^{\sigma}). \tag{9}$$

Calculation results are shown in Fig.1 (see panel (b)). Comparison with the results obtained without Coulomb interaction reveals the shift of the tunneling conductance spectra and the appearance of the "shoulder" in the tunneling conductance for the positive values of applied bias voltage. It is directly the result of the tunneling conductance modification by the occupation numbers which are renormalised by the presence of Coulomb interaction.

3 Occupation numbers when electron transport occurs through two parallel channels with an impurity atom each

Let us evaluate occupation numbers in the situation when electron transport occurs through two parallel channels each of them containing an impurity atom. Equations, which determine occupation numbers can be written using Green's functions formalism:

$$\begin{aligned}
0 &= \frac{\partial}{\partial t}G_{11}^{<} = T(G_{21}^{<}-G_{12}^{<})+2\Gamma_{L1}ImG_{11}^{R}(n_1-n_p^0)+2\Gamma_{R1}ImG_{11}^{R}(n_1-n_k^0),\\
0 &= \frac{\partial}{\partial t}G_{22}^{<} = T(G_{12}^{<}-G_{21}^{<})+2\Gamma_{L2}ImG_{22}^{R}(n_2-n_p^0)+2\Gamma_{R2}ImG_{22}^{R}(n_2-n_k^0),\\
0 &= \frac{\partial}{\partial t}G_{12}^{<} = -R_{12}^{-1}G_{12}^{<}+T(G_{22}^{<}-G_{11}^{<})+2(\Gamma_{L1}G_{12}^{A}-\Gamma_{L2}G_{12}^{R})n_p^0+2(\Gamma_{R1}G_{12}^{A}-\Gamma_{R2}G_{12}^{R})n_k^0,\\
0 &= \frac{\partial}{\partial t}G_{21}^{<} = -R_{12}^{-1}G_{21}^{<}+T(G_{11}^{<}-G_{22}^{<})+2(\Gamma_{L2}G_{21}^{A}-\Gamma_{L1}G_{21}^{R})n_p^0-2(\Gamma_{R1}G_{21}^{R}-\Gamma_{R2}G_{21}^{A})n_k^0,
\end{aligned} \tag{10}$$

where

$$\begin{aligned}
R_{12} &= \frac{1}{\varepsilon_1-\varepsilon_2+i(\Gamma_1+\Gamma_2)},\\
G_{12}^{R} &= \frac{T}{(\omega-\varepsilon_1+i\Gamma_1)(\omega-\varepsilon_2+i\Gamma_2)-T^2},\\
G_{11}^{R} &= \frac{\omega-\varepsilon_2+i\Gamma_2}{(\omega-\varepsilon_1+i\Gamma_1)(\omega-\varepsilon_2+i\Gamma_2)-T^2},\\
G_{22}^{R} &= \frac{\omega-\varepsilon_1+i\Gamma_1}{(\omega-\varepsilon_1+i\Gamma_1)(\omega-\varepsilon_2+i\Gamma_2)-T^2}.
\end{aligned} \tag{11}$$

System of equations (10) gives the following equations for occupation numbers:

$$\begin{aligned}
n_1ImG_{11}^{R}(\omega)\Gamma_1+n_2ImG_{22}^{R}(\omega)\Gamma_2 &= ImG_{11}^{R}(\omega)(\Gamma_{L1}n_p^0+\Gamma_{R1}n_k^0)+ImG_{22}^{R}(\omega)(\Gamma_{L2}n_p^0+\Gamma_{R2}n_k^0),\\
\eta(n_1ImG_{11}^{R}(\omega)-n_2ImG_{22}^{R}(\omega)) &= \Gamma_{L2}ImG_{22}^{R(\omega)}(n_2-n_p^0)+\Gamma_{L1}ImG_{11}^{R}(\omega)(n_1-n_k^0)+\\
+n_p^0T[\Gamma_{L1}Im(R_{12}(\omega)G_{12}^{A}(\omega))-\Gamma_{L2}Im(R_{12}(\omega)G_{12}^{R}(\omega))] &+ n_k^0T[\Gamma_{R1}Im(R_{12}(\omega)G_{12}^{A}(\omega))-\Gamma_{R2}Im(R_{12}(\omega)G_{12}^{R}(\omega))]
\end{aligned} \tag{12}$$

with

$$\eta = T^2\frac{\Gamma_1+\Gamma_2}{(\varepsilon_1-\varepsilon_2)^2+(\Gamma_1+\Gamma_2)^2}. \tag{13}$$

Integrating Eqs.(12) over ω one can get system of linear equations which determine occupation numbers of impurities in the limit of strong Coulomb interaction. Substituting the solution of the linear system of equations to the Green's functions of impurity states corresponding to the limit of large Coulomb interaction ($G_s^R=\frac{1-n_1-2n_2}{\omega-\varepsilon_1+i\Gamma_1}$ and $G_S^a=\frac{1-n_2-2n_1}{\omega-\varepsilon_2+i\Gamma_2}$) and using expression (13) in the case of two impurities one can calculate tunneling conductance in the presence of Coulomb interaction.

References

1. Fano, U. Effects of configuration interaction on intensities and phase shifts. *Phys. Rev.* **124**, 1866–1878, DOI: 10.1103/PhysRev.124.1866 (1961).
2. Maslova, N., Arseyev, P. & Mantsevich, V. Correlated impurity complex in the asymmetric tunneling contact: an ideal system to observe negative tunneling conductivity. *Sci. Reports* **9**, 15974, DOI: 10.1038/s41598-019-52095-8 (2019).
3. Maslova, N., Arseyev, P. & Mantsevich, V. Control of the non-stationary spin-polarized tunneling currents by applied bias changing. *Solid State Commun.* **248**, 21–26, DOI: https://doi.org/10.1016/j.ssc.2016.09.003 (2016).
